# Reliability of major bleeding events in UK routine data versus clinical trial adjudicated follow-up data

**DOI:** 10.1136/heartjnl-2023-322616

**Published:** 2023-06-03

**Authors:** Charlie Harper, Marion Mafham, William Herrington, Natalie Staplin, William Stevens, Karl Wallendszus, Richard Haynes, Martin J Landray, Sarah Parish, Louise Bowman, Jane Armitage

**Affiliations:** 1 Medical Research Council Population Health Research Unit at the University of Oxford, Nuffield Department of Population Health (NDPH), University of Oxford, Oxford, UK; 2 Clinical Trial Service Unit and Epidemiological Studies Unit (CTSU), NDPH, University of Oxford, Oxford, UK; 3 Big Data Institute, Li Ka Shing Centre for Health Information & Discovery, NDPH, University of Oxford, Oxford, UK

**Keywords:** electronic health records, atherosclerosis, outcome assessment, health care, research design

## Abstract

**Objective:**

To assess how reliable UK routine data are for ascertaining major bleeding events compared with adjudicated follow-up.

**Methods:**

The ASCEND (A Study of Cardiovascular Events iN Diabetes) primary prevention trial randomised 15 480 UK people with diabetes to aspirin versus matching placebo. The primary safety outcome was major bleeding (including intracranial haemorrhage, sight-threatening eye bleeding, serious gastrointestinal bleeding and other major bleeding (epistaxis, haemoptysis, haematuria, vaginal and other bleeding)) ascertained by direct-participant mail-based follow-up, with >90% of outcomes undergoing adjudication. Nearly all participants were linked to routinely collected hospitalisation and death data (ie, routine data). An algorithm categorised bleeding events from routine data as major/minor. Kappa statistics were used to assess agreement between data sources, and randomised comparisons were re-run using routine data.

**Results:**

When adjudicated follow-up and routine data were compared, there was agreement for 318 major bleeding events, with routine data identifying 281 additional-potential events, and not identifying 241 participant-reported events (kappa 0.53, 95% CI 0.49 to 0.57). Repeating ASCEND’s randomised comparisons using routine data only found estimated relative and absolute effects of allocation to aspirin versus placebo on major bleeding similar to adjudicated follow-up (adjudicated follow-up: aspirin 314 (4.1%) vs placebo 245 (3.2%); rate ratio (RR) 1.29, 95% CI 1.09 to 1.52; absolute excess +6.3/5000 person-years (mean SE±2.1); vs routine data: 327 (4.2%) vs 272 (3.5%); RR 1.21, 95% CI 1.03 to 1.41; absolute excess +5.0/5000 (±2.2)).

**Conclusions:**

Analyses of the ASCEND randomised trial found that major bleeding events ascertained via UK routine data sources provided relative and absolute treatment effects similar to adjudicated follow-up.

**Trial registration number:**

ISRCTN60635500; NCT00135226.

**WHAT IS ALREADY KNOWN ON THIS TOPIC**

- Linkage to routinely collected healthcare data sources could substantially simplify randomised trial follow-up by offering a cost-efficient method to ascertain outcomes. Previous analyses have demonstrated the utility of using such data sources in the UK for vascular outcomes.

**WHAT THIS STUDY ADDS**

- By applying an algorithm to UK routinely collected hospitalisation and death registry data, it may be feasible to categorise bleeding events as major or minor.
- Randomised comparisons using routine data only found estimated relative and absolute effects of allocation to aspirin versus placebo on major bleeding similar to adjudicated follow-up.
- If all bleeding events recorded in the routine data had been considered major, the significant bleeding hazard would have been missed.

**HOW THIS STUDY MIGHT AFFECT RESEARCH, PRACTICE OR POLICY**

- UK routine data represent a potentially cost-efficient method to ascertain major bleed trial outcomes during both within and post-trial periods, either to supplement existing methods or to be used on its own.

## Introduction

Large randomised trials in cardiovascular disease have provided reliable evidence for interventions whose widespread use has contributed to declines in mortality due to cardiovascular disease.^1^ However, increasing costs and complexity of conducting trials threaten our ability to generate new randomised evidence.^2^ Linkage to routinely collected healthcare data sources could substantially simplify trial follow-up by offering a cost-efficient method to ascertain outcomes.^3^ A Study of Cardiovascular Events iN Diabetes (ASCEND),^4–6^ a 15 480 participant mail-based double-blind placebo-controlled randomised trial in people with diabetes but no evidence of atherosclerotic vascular disease at recruitment, was conducted in the UK from 2005 to 2017. Previous analyses using ASCEND data linked to routinely collected UK hospitalisation and death registry data demonstrated the utility of using such data sources to follow-up trial participants for serious vascular outcomes.^7^ Major bleeding was the primary safety outcome for ASCEND’s aspirin versus matching placebo randomised comparison. This study aimed to use the ASCEND-linked data to assess whether, compared with adjudicated direct-participant follow-up, UK routine data can reliably follow-up participants for major bleeding outcomes in future streamlined trials by (1) measuring agreement between data sources, and (2) re-running the aspirin versus placebo randomised comparisons to estimate both the relative and absolute bleeding risk using the hypothetical scenario that follow-up had been solely through linkage to routine data. As a subsidiary aim, the impact of adjudication on major bleeding outcomes ascertained from mail-based direct-participant follow-up was assessed.

## Methods

### ASCEND trial

Full details of the ASCEND trial design (online supplemental file 1) and its findings have been reported previously.^4–6^ Briefly, between 2005 and 2011, 15 480 UK participants were randomised using a 2×2 factorial design to 100 mg of aspirin once daily versus matching placebo, and separately to 1 g capsules containing omega-3 fatty acids once daily versus placebo. For the aspirin comparison (online supplemental figure 1), the primary efficacy assessment was time to first serious vascular event (SVE; a composite of non-fatal myocardial infarction, presumed ischaemic stroke or transient ischaemic attack, and vascular death (excluding intracranial haemorrhage)). The primary safety assessment was time to first major bleeding event (a composite of any intracranial haemorrhage, sight-threatening bleeding in eye, serious gastrointestinal bleeding (including upper, lower or unspecified bleeding, and perforation) and other major bleeding (including epistaxis, haemoptysis, haematuria, vaginal and other bleeding)). For gastrointestinal or other bleeding events to meet the ASCEND criteria of major bleeding, they would have either led to hospitalisation or death; full details of the bleeding subcategorisation used in ASCEND are provided in online supplemental figure 2. Sight-threatening eye bleeds included any clinically significant retinal bleeds presenting with symptoms outside of routine retinopathy screening which required laser photocoagulation, surgery or intraocular injections or any intraocular bleeding resulting in permanent visual loss. All intracranial bleeds were considered to be major. Mean follow-up was 7.4 years (about 114 000 person-years).

10.1136/heartjnl-2023-322616.supp1 Supplementary data

10.1136/heartjnl-2023-322616.supp3 Supplementary data

### Direct-participant mail-based follow-up and adjudication

The principal method of direct-participant recruitment and follow-up was by mailed or online questionnaire 6-monthly. On questionnaires, participants were asked to indicate if they had suffered ‘Bleeding for which you saw a doctor’ and to record a date and the site of the bleeding. Participants were also asked ‘Were you admitted to hospital?’ Study clinicians blind to treatment allocation coded the bleeding site using the free-text information, and these data are referred to in this manuscript as ‘pre-adjudicated direct-participant follow-up’ (online supplemental figure 3). Study clinicians blind to treatment allocation adjudicated >90% of the primary and secondary outcomes (for further details about the adjudication process in the ASCEND trial, see online supplemental methods). These outcome data were used in ASCEND’s primary publications^5 6^ and are referred to as ‘adjudicated direct-participant follow-up’ in this manuscript (online supplemental figure 3).

10.1136/heartjnl-2023-322616.supp2 Supplementary data

### Routinely collected death records and hospital admission data

Consent was obtained from participants to allow access to their routinely collected data. During follow-up, routinely collected death records and hospitalisation data were obtained for participants living in England, Scotland and Wales, but was not possible for 44 participants (44/15480 (0.3%)) in Northern Ireland (for further details about the routine data sources used, see online supplemental methods). Bleeding episodes were identified in death records and hospital admission data using clinician specified International Classification of Diseases (ICD-10) diagnosis codes (online supplemental table 1). The date of event was assumed to be the date of admission or death. The bleeding code must have been recorded as the underlying cause on the death record to be considered fatal (online supplemental table 2). For bleeding codes recorded in the hospital admissions data, 15 possible algorithms were developed to categorise the bleeding events as major or minor, with one selected for the primary analyses by clinicians before any unbinding (see online supplemental methods). For any intracranial haemorrhage or serious eye bleed, the selected algorithm defined this as an ICD-10 code in any diagnostic position (ie, primary or secondary diagnosis), while for gastrointestinal or other bleeding to be classified as major, records were restricted to bleeding codes in the first (ie, primary) diagnostic position and patients having stayed at least one night in hospital (online supplemental table 2). Previously published methods were used to ascertain SVEs (online supplemental table 3).^7^ Events identified through routinely collected data did not undergo clinical adjudication and are referred to as ‘routine data’ (online supplemental figure 3).

### Statistical analyses

Analyses included only those events that occurred between randomisation and date of death or censoring, except for participants living in Northern Ireland where routine data follow-up was censored at day zero. For the major bleeding outcome (and its components), participants were categorised as having ‘outcome in both datasets’, ‘outcome in routine data only’, ‘outcome in adjudicated follow-up alone’ or ‘no such outcome in either dataset’, irrespective of the relative timings of the events. Comparisons between routine data and adjudicated direct-participant follow-up were performed using sensitivity, specificity and kappa statistics with 95% CIs.^8 9^ Details about further sensitivity analyses can be found in online supplemental methods.

All randomised comparisons used standard log-rank methods^10 11^ following the intention-to-treat approach. Rate ratios (RRs) with 95% CI are provided. The main randomised comparisons were those based on the adjudicated direct-participant follow-up data versus those based on the alternative scenario that ASCEND had only used routine data to identify outcomes. Differences between the RRs were calculated, with 95% CI derived using bootstrap methods. Observed absolute treatment effects were also calculated in which results were presented as number of events per 5000 person-years including the mean SE. Sensitivity analyses included re-running randomised comparisons using the 14 other proposed algorithms to distinguish major from minor bleeding. Secondarily, the impact of adjudication on randomised comparisons was assessed using outcomes derived from pre-adjudicated direct-participant follow-up. Analyses were conducted using SAS V.9.4 (SAS Institute) and R V.4.1.1 (R Project for Statistical Computing).

## Results

### Reliability of routine data

Of the 1690 participants with a bleeding event identified from routine data sources, 599 (35.4%) were categorised as major by the routine data algorithm (online supplemental figure 3). Adjudicated direct-participant follow-up identified 559 participants with a major bleeding event, of which 318 also had a major bleeding event recorded in the routine data (table 1). Routine data identified an additional 281 potential major bleeding events not previously reported by adjudicated direct-participant follow-up. Overall agreement between routine data and adjudicated direct-participant follow-up for the any major bleeding outcome was moderate (kappa 0.53, 95% CI 0.49 to 0.57), with similar kappa statistics across subgroups of participants (online supplemental table 4). For the 318 participants with a major bleeding event in adjudicated direct-participant follow-up and routine data, date of first recorded major bleeding event in both data sources were within 90 days of each other for 260 (81.8%) participants (online supplemental table 5). Agreement statistics for the alternative 14 major bleeding routine data algorithms not pre-selected for analysis can be found in online supplemental table 6.

**Table 1:** Agreement between routine data and adjudicated direct follow-up

Outcome	Outcome in both datasets	Outcome in routine data only	Outcome in adjudicated follow-up alone	No such outcome in either dataset	Sensitivity (95% CI)	Specificity (95% CI)	Kappa (95% CI)
Intracranial haemorrhage	87 (0.6%)	50 (0.3%)	13 (0.1%)	15 330 (99.0%)	87.0% (80.4% to 93.6%)	99.7% (99.6% to 99.8%)	0.73 (0.67 to 0.80)
Sight-threatening bleeding in eye	49 (0.3%)	48 (0.3%)	72 (0.5%)	15 311 (98.9%)	40.5% (31.7% to 49.2%)	99.7% (99.6% to 99.8%)	0.45 (0.35 to 0.54)
Serious gastrointestinal bleeding	131 (0.8%)	96 (0.6%)	107 (0.7%)	15 146 (97.8%)	55.0% (48.7% to 61.4%)	99.4% (99.2% to 99.5%)	0.56 (0.50 to 0.62)
Other major bleeding	51 (0.3%)	113 (0.7%)	66 (0.4%)	15 250 (98.5%)	43.6% (34.6% to 52.6%)	99.3% (99.1% to 99.4%)	0.36 (0.26 to 0.45)
**Any major bleeding**	**318** (**2.1%**)	**281** (**1.8%**)	**241** (**1.6%**)	**14 640** (**94.6%**)	**56.9% (52.8% to 61.0%**)	**98.1% (97.9% to 98.3%**)	**0.53 (0.49 to 0.57**)

Percentages in parentheses are % of total number of ASCEND participants. Sensitivity and specificity statistics calculated using adjudicated direct follow-up as the reference dataset.

For the components of the major bleeding composite outcome, agreement between adjudicated direct-participant follow-up and routine data was strongest for intracranial haemorrhage (kappa 0.73, 95% CI 0.67 to 0.80) and serious gastrointestinal bleeding (0.56, 95% CI 0.50 to 0.62; online supplemental table 7). Other major bleeding and sight-threatening eye bleeding had poor (0.36, 0.26 to 0.45) and moderate agreement (0.45, 0.35 to 0.45), respectively.

For the 241 major bleeding events identified in adjudicated direct-participant follow-up alone, within 90 days, 74 (30.7%) had a bleeding code recorded in the routine data but did not meet the algorithm’s definition for major bleeding, 93 (38.6%) had a routine data hospitalisation record but no bleeding code recorded and 74 (30.7%) had no routine data hospitalisation record (online supplemental table 8). For the 281 major bleeding events identified in routine data only, in the adjudicated direct-participant follow-up database, 11 (3.9%) participants had a bleed event within 90 days which was refuted after adjudication, 10 (3.6%) had a minor bleed reported and 223 (79.4%) had no hospitalisation reported within 90 days.

### Randomised comparisons using routine data

**Figure 1:**
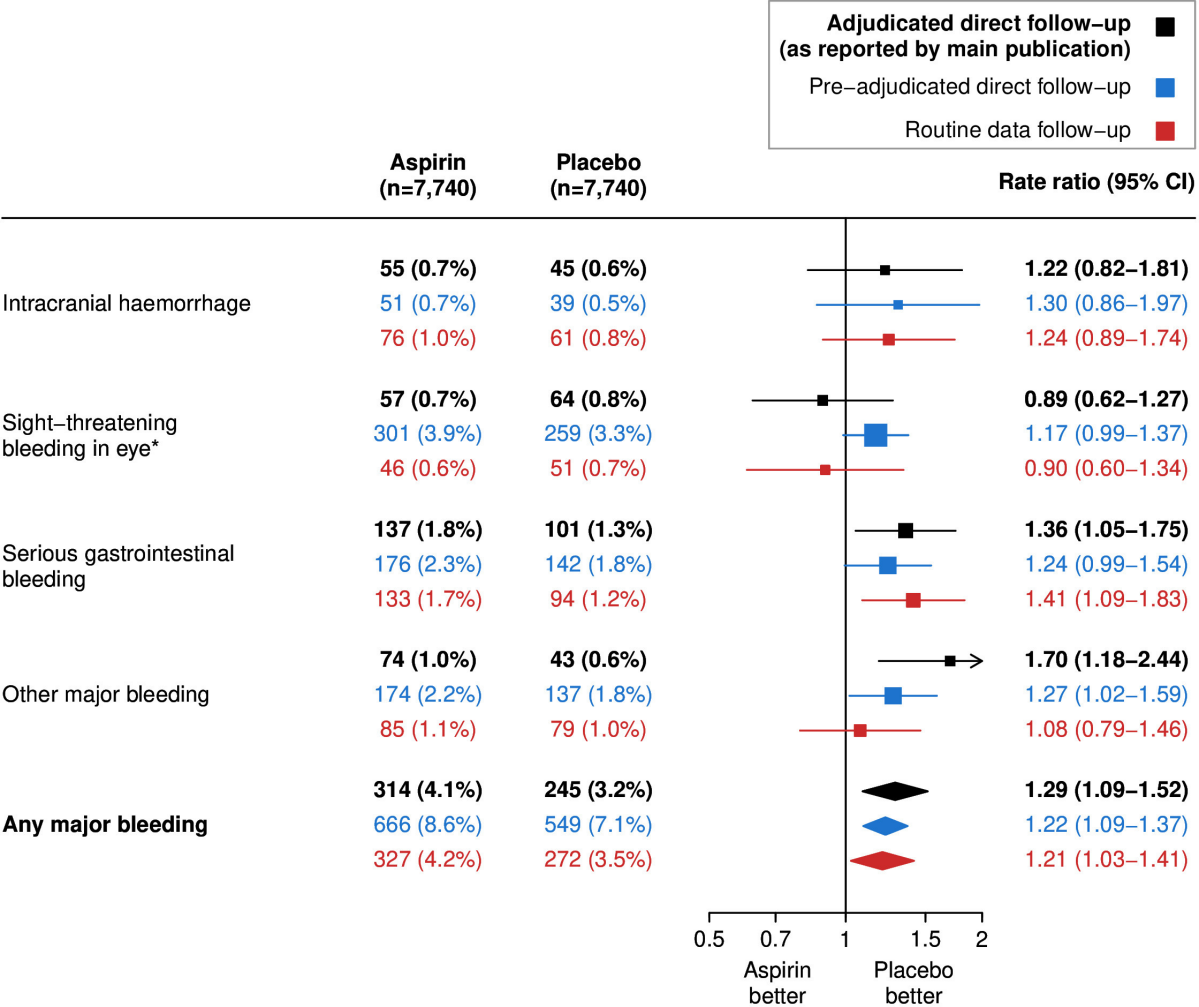
Effect of allocation to aspirin vs placebo on any major bleeding. Log-rank methods were used to calculate the rate ratio and 95% CIs. *The pre-adjudicated direct follow-up outcome included all eye bleeds.

For the any major bleeding outcome, there were no important differences between estimates of the relative effects of aspirin versus placebo using routine data alone and published results based on adjudicated direct-participant follow-up (adjudicated direct-participant follow-up: aspirin 314 (4.1%) versus placebo 245 (3.2%); RR 1.29, 95% CI 1.09 to 1.52; versus routine data: 327 (4.2%) versus 272 (3.5%); RR 1.21, 95% CI 1.03 to 1.41; difference in point estimate: −0.08, 95% CI −0.28 to +0.12; figure 1 and online supplemental table 9). When routine data follow-up was broken down by data source, events identified in routine data only were found to have a lower RR than events identified by both data sources (online supplemental table 10). Randomised comparisons performed using the 14 routine data algorithms not pre-selected for analyses prior to unblinding found that the fewer severity factors included in the algorithm, the lower the relative effects of aspirin, and that the major bleeding hazard would have been missed if we had considered all bleeding events recorded in the routine data as major (RR 1.05, 95% CI 0.96 to 1.16; online supplemental figure 4 and online supplemental table 11).

**Figure 2:**
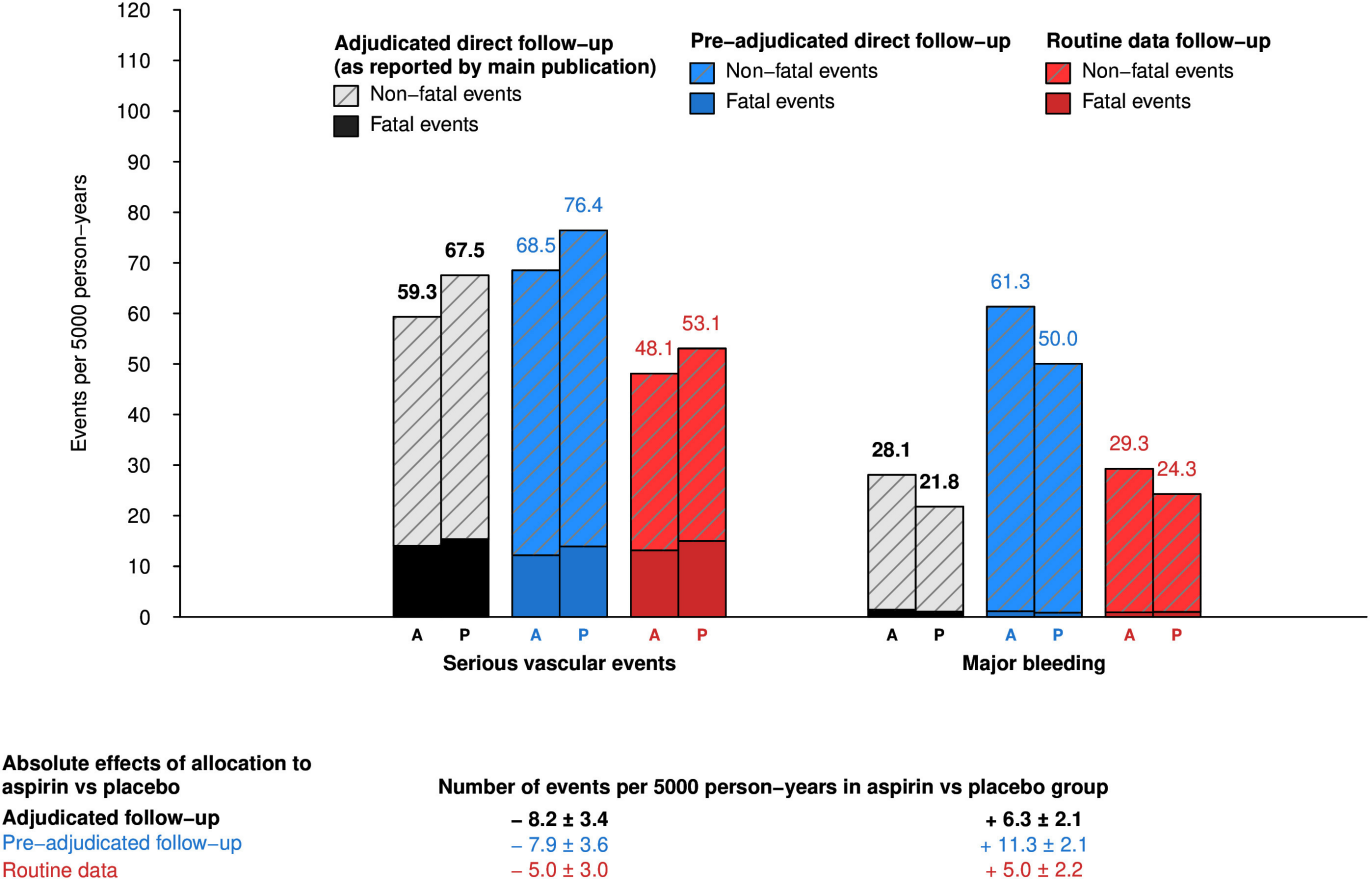
Observed absolute effects of allocation to aspirin (A) vs placebo (P) for routine data follow-up. Data expressed as numbers of events per 5000 person-years. Plus-minus value are mean SEs. Serious vascular events include non-fatal myocardial infarction, ischaemic stroke, transient ischaemic attack or vascular death (excluding intracranial haemorrhage).

Figure 2 provides estimates of the observed absolute effects of allocation to aspirin versus placebo for the SVE and major bleeding outcomes. Similar to adjudicated direct-participant follow-up (SVE −8.2 per 5000 person-years (mean SE±3.4) vs major bleeding +6.3 (±2.1)), when events were ascertained solely through routine data, the absolute benefits of aspirin were largely counterbalanced by the bleeding hazard (SVE −5.0 (±3.0) vs major bleeding +5.0 (±2.2)).

### Impact of adjudication on mail-based direct-participant follow-up

Of the 1215 major bleeds reported via mail-based direct-participant follow-up, 58.4% (710) were refuted during adjudication, with 496 being considered to be minor bleeds and 214 not being considered a bleeding event (online supplemental table 12). These results differed by bleeding type: adjudication confirmed 92.2% (83/90) of direct-participant reported intracranial haemorrhages and just over two-thirds (69.5% (221/318)) of reported serious gastrointestinal bleeding events (table 2). For subcomponents of gastrointestinal bleeding, adjudication confirmation rates were far higher for upper (83.3% (135/162)) compared with lower gastrointestinal bleeding (47.6% (71/149); online supplemental table 13). The confirmation rates were low for other major bleeding (30.2% (94/311); online supplemental table 14), and lowest for sight-threatening eye bleeding (16.8% (94/560)), which was expected since pre-adjudication direct-participant reports included all eye bleeds not just those considered sight threatening. For the any major bleeding outcome, there were no important differences in the estimated relative effects of allocation to aspirin versus placebo between pre-adjudicated and adjudicated direct-participant follow-up (pre-adjudicated direct-participant follow-up: aspirin 666 (8.6%) vs placebo 549 (7.1%); RR 1.22, 95% CI 1.09 to 1.37; figure 1), while the absolute effects were almost double that of adjudicated direct-participant follow-up (pre-adjudicated direct-participant follow-up: +11.3 per 5000 person-years (mean SE±2.1); figure 2).

**Table 2:** Comparison between pre-adjudicated and post-adjudicated direct follow-up

Bleeding category before adjudication	Bleeding category after adjudication						
	Intracranial haemorrhage	Sight-threatening bleeding in eye	Serious gastrointestinal bleeding	Other major bleeding	Minor bleeding	No bleeding	Total
Intracranial haemorrhage	**83** (**92.2%**)	0 (0.0%)	0 (0.0%)	0 (0.0%)	0 (0.0%)	7 (7.8%)	**90**
Any eye bleeding	3 (0.5%)	**94** (**16.8%**)	0 (0.0%)	0 (0.0%)	248 (44.3%)	215 (38.4%)	**560**
Serious gastrointestinal bleeding	1 (0.3%)	0 (0.0%)	**221** (**69.5%**)	4 (1.3%)	65 (20.4%)	27 (8.5%)	**318**
Other major bleeding	2 (0.6%)	0 (0.0%)	3 (1.0%)	**94** (**30.2%**)	190 (61.1%)	22 (7.1%)	**311**
Total*	**100**	**121**	**238**	**117**			

Percentages in parentheses are % of total number of ASCEND participants with the bleeding category before adjudication. The total (for each row or column) counts only the first event that occurred for each participant.

*The sum of each column does not equal the total because a small number of minor and non-bleeding events were categorised as major bleeding events after adjudication (see online supplemental table 11).

## Discussion

In these analyses from the ASCEND^4–6^ randomised trial, compared with adjudicated direct-participant follow-up, we found that by applying an algorithm to UK routinely collected hospitalisation and death registry data, major bleeding events (a composite of any intracranial haemorrhage, sight-threatening bleeding in the eye, serious gastrointestinal bleeding and other major bleeding) identified using routine data follow-up provided a similar estimate of the relative effects of aspirin versus placebo. The interpretation of ASCEND’s aspirin randomised comparison (ie, the benefits of aspirin being largely counterbalanced by the bleeding hazard) would have not changed had follow-up been solely via routinely collected data, but when no algorithm was applied (ie, when all bleeding events recorded in the routine data were considered major), the significant bleeding hazard would have been missed. For the components of the major bleeding outcome, intracranial haemorrhage showed strongest agreement, presumably because these events were the most serious. These important results complement findings from ASCEND’s analyses of serious vascular outcomes^7^ which also suggested that UK routine data represent a potentially cost-efficient method to ascertain trial outcomes during both within and post-trial periods.

For major bleeding events, ASCEND is one of the first published studies to compare relative and absolute treatment effects from a large-scale randomised trial using adjudicated direct follow-up versus the hypothetical scenario that follow-up had been solely through routinely collected data. A previous study assessing routine data ascertained major bleeding events in England^12 13^ concluded that, as the ICD-10 coding system does not stratify bleeds into degrees of severity, routine hospital admission data are of limited utility to randomised trials. However, our findings suggest that, by developing an algorithm using potential indicators of severity available in the routine data, it may be possible to reliably subcategorise bleeding events into major and minor. Similarly, in the USA, by using a validated algorithm,^14^ the ADAPTABLE trial^15^ was able to follow up participants almost solely via routine data sources for their primary safety outcome of major bleeding, without the need for verification by clinical adjudication.

Our analysis of the ASCEND trial highlights the challenges of ensuring complete follow-up for major bleeding outcomes when using either adjudicated direct-participant mail-based follow-up or routinely collected data. When comparing these two methods of follow-up, we found that although there was moderate agreement, over 200 major bleeding events were either reported in adjudicated direct-participant follow-up alone or routine data only. Importantly, in ASCEND, despite these discrepancies, relative and absolute treatment effects were similar between both data sources. However, if a trial must ensure complete follow-up for major bleeding outcomes, then this will likely require a combined approach of direct participant and routine data follow-up.

For the major bleeding events only identified in adjudicated direct-participant follow-up, the majority had a corresponding routinely collected hospitalisation record, but either the record gave no indication of bleeding severity or no bleeding code was recorded. Although there has been an expansion in sources of routine data available for UK clinical trials,^16 17^ secondary care datasets that could help categorise bleeding events are not yet collected nationally.^18^ Expanding existing hospital data flows to also include within-hospital prescribing, laboratory results and standardised patient discharge summaries could improve our algorithm’s performance at ascertaining major bleeding events from routine data.

A number of studies have reported on the impact of clinical adjudication on investigator-reported and participant-reported cardiovascular events.^7 19–22^ However, the accuracy of mail-based direct-participant follow-up reported major bleeding events compared with clinical adjudication has not been previously reported. Despite the fact that over half of the pre-adjudicated mail-based direct-participant reported bleed events were refuted by the adjudication process, the relative effects of aspirin on pre-adjudicated outcomes was only slightly lower than the effects on adjudicated outcomes. However, the absolute effects of aspirin were found to be materially different, with pre-adjudicated direct-participant follow-up providing an absolute bleeding hazard almost double that of the adjudicated data. Thus, clinical adjudication of mail-based direct-participant follow-up may still be necessary in order to adequately weigh up the absolute harms and benefits of antiplatelet therapy.

There are some limitations of the current analyses. First, the ASCEND mail-based follow-up reports appeared to miss some major bleeding events and did not have the associated medical notes to verify or refute major bleeds identified in routine data only; thus, there are challenges to interpreting the agreement statistics presented. Second, ASCEND used innovative mail-based methods, so comparisons with traditional methods of site-investigator reported major bleeding were not possible. Third, findings are based on the particular definition of major bleeding used in ASCEND; these definitions were an adaptation of the Bleeding Academic Research Consortium (BARC)^23^ and are still likely to be widely relevant. But as much of the BARC criteria cannot be ascertained from current UK source of routine data (eg, drops in haemoglobin), ASCEND’s more pragmatic major bleeding definition may be more appropriate for future streamlined trials. Fourth, participants in ASCEND had a mean follow-up of 7.4 years, and routine data may have greater utility in long-term chronic disease trials such as these, compared with trials of acute interventions where follow-up may be limited to a number of weeks meaning that precise timing of outcomes is important.^13^ Finally, the ASCEND trial was carried out in the UK which has a national health service; the study therefore was not able to assess the reliability of routine data in non-UK countries, including those that do not have a single national healthcare provider nor if trials are conducted across different countries.

## Conclusion

In summary, these analyses of the ASCEND randomised trial found that major bleeding events ascertained via applying an algorithm to UK routinely collected hospital admission and death registry data provided relative and absolute treatment effects similar to adjudicated direct participant follow-up.

These results suggest that UK routine data represent a potentially cost-efficient method to ascertain major bleed trial outcomes during both within and post-trial periods, either to supplement existing methods or to be used on its own. However, further work is needed to validate this algorithm in other trial populations. Finally, our study found that clinical adjudication of mail-based direct-participant follow-up may still be necessary in order to adequately weigh up the absolute harms and benefits of antiplatelet therapy.

## Data Availability

No data are available. Post-trial follow-up of the ASCEND cohort is ongoing using the routinely collected data, with planned analyses 5 and 10 years after the end of the scheduled treatment period. Data sharing will be considered in line with the Nuffield Department of Population Health, University of Oxford, Data Access and Sharing Policy (available at https://www.ndph.ox.ac.uk/about/data-access-policy).
