# Supplementary material for: Reliability of major bleeding events in UK routine data versus clinical trial adjudicated follow-up data
Source: Heart. 2023 Jun 3;109(19):1467–72. doi: 10.1136/heartjnl-2023-322616 (PMC10511984; doi:10.1136/heartjnl-2023-322616)
Supplement: Supplementary data [file heartjnl-2023-322616supp003.pdf]

Supplemental Tables and Figures

Supplemental table 1: Codes used to identify any bleeding events in routine data

Supplemental table 2: Algorithm used to categorise major bleeding events in routine data

Supplemental table 3: Codes used to identify any serious vascular events in routine data

Supplemental table 4: Agreement of routine data versus adjudicated direct follow-up for any major bleeding, by subgroups

Supplemental table 5: Comparison of routine data event date versus adjudicated direct follow-up

Supplemental table 6: Agreement of routine data versus adjudicated direct follow-up, by routine data algorithm

Supplemental table 7: Agreement of routine data versus adjudicated direct follow-up, by subcomponents of the major bleeding outcome

Supplemental table 8: Major bleeding events identified in adjudicated direct follow-up alone or routine data only

Supplemental table 9: Comparison of estimated treatment effects between adjudicated direct follow-up and routine data follow-up for the aspirin randomised comparison

Supplemental table 10: Effect of allocation to aspirin versus placebo on any major bleeding, by source of outcome data

Supplemental table 11: Effect of allocation to aspirin versus placebo on serious gastrointestinal and other major bleeding, by routine data algorithm

Supplemental table 12: Comparison of bleeding categories between pre and post adjudicated direct follow-up

Supplemental table 13: Comparison of bleeding categories between pre and post adjudicated direct follow-up, by components of the serious gastrointestinal bleeding outcome

Supplemental table 14: Comparison of bleeding categories between pre and post adjudicated direct follow-up, by components of the other major bleeding outcome

Supplemental figure 1: Flow diagram of post hoc analyses in the ASCEND trial

Supplemental figure 2: Bleeding severity criteria for ASCEND adjudicated direct follow-up

Supplemental figure 3: Sources of major bleeding events in ASCEND

Supplemental figure 4: Effect of allocation to aspirin versus placebo on any major bleeding, by routine data algorithm

Harper C, *et al. Heart* 2023; 109:1467–1472. doi: 10.1136/heartjnl-2023-322616

Supplemental table 1: Codes used to identify any bleeding events in routine data

Outcome	ICD-10 codes
**Any bleeding**	
Intracranial haemorrhage	Subarachnoid haemorrhage (I60); intracerebral haemorrhage (I61); other intracranial haemorrhage (I62); traumatic subdural haemorrhage (S06.5); traumatic subarachnoid haemorrhage (S06.6)
Eye bleed	Choroidal haemorrhage and rupture (H31.3); retinal haemorrhage (H35.6); vitreous haemorrhage (H43.1); vitreous haemorrhage in diseases classified elsewhere (H45.0)
Gastrointestinal bleeding	
Upper bleeding	Oesophageal varices, with bleeding (I85.0); gastro-oesophageal laceration-haemorrhage syndrome (K22.6); gastric ulcer, acute with haemorrhage (K25.0), acute with both haemorrhage and perforation (K25.2), chronic or unspecified with haemorrhage (K25.4), or chronic/unspecified with both haemorrhage or perforation (K25.6); duodenal ulcer, acute with haemorrhage (K26.0), acute with both haemorrhage and perforation (K26.2), chronic or unspecified with haemorrhage (K26.4), or chronic/unspecified with both haemorrhage or perforation (K26.6); peptic ulcer, acute with haemorrhage (K27.0), acute with both haemorrhage and perforation (K27.2), chronic or unspecified with haemorrhage (K27.4), or chronic/unspecified with both haemorrhage or perforation (K27.6); gastrojejunial ulcer, acute with haemorrhage (K28.0), acute with both haemorrhage and perforation (K28.2), chronic or unspecified with haemorrhage (K28.4), or chronic/unspecified with both haemorrhage or perforation (K28.6); acute haemorrhagic gastritis (K29.0); hematemesis (K92.0); melaena (K92.1)
Lower bleeding	Haemorrhage of anus and rectum (K62.5)
Unspecified bleeding	Gastrointestinal haemorrhage, unspecified (K92.2)
Perforation	Perforation of oesophagus (K22.3), or intestine (K63.1); gastric ulcer, acute with perforation (K25.1), or chronic/unspecified with perforation (K25.5); duodenal ulcer, acute with perforation (K26.1), or chronic/unspecified with perforation (K26.5); peptic ulcer, acute with perforation (K27.1), or chronic/unspecified with perforation (K27.5); gastrojejunial ulcer, acute with perforation (K28.1), or chronic/unspecified with perforation (K28.5)
Other bleeding	
Epistaxis	Epistaxis (R04.0)
Haemoptysis	Haemoptysis (R04.2)
Haematuria	Unspecified haematuria (R31); recurrent and persistent haematuria (N02); congestion and haemorrhage of prostate (N42.1)
Vaginal bleeding	Excessive and frequent menstruation with irregular cycle (N92.1); other specified irregular menstruation (N92.5); irregular menstruation, unspecified (N92.6); other abnormal uterine and vaginal bleeding (N93); postmenopausal bleeding (N95.0)
Unspecified bleeding	Haemorrhage, not elsewhere classified (R58); traumatic secondary and recurrent haemorrhage (T79.2); haemorrhage and hematoma complicating a procedure, not elsewhere classified (T81.0)

ICD-10 = International Statistical Classification of Diseases and Related Health Problems (tenth revision).

Harper C, *et al. Heart* 2023; 109:1467–1472. doi: 10.1136/heartjnl-2023-322616

Supplemental table 2: Algorithm used to categorise major bleeding events in routine data

Outcome	Routine data algorithm
**Any major bleeding**	
Intracranial haemorrhage	Hospital admission with an intracranial haemorrhage code recorded in any diagnostic position; OR death record where intracranial haemorrhage code recorded as the underlying cause
Serious eye bleed	Hospital admission with an eye bleed code recorded in any diagnostic position
Serious gastrointestinal bleeding*	Hospital admission with a gastrointestinal bleeding code recorded in the primary diagnostic position AND the participant stayed at least one night in hospital; OR death record where gastrointestinal bleeding code recorded as the underlying cause
Other major bleeding	Hospital admission with other bleeding code recorded in the primary diagnostic position AND the participant stayed at least one night in hospital; OR death record where other bleeding code recorded as the underlying cause

*For unspecified gastrointestinal bleeding, if during the same admission an upper gastrointestinal procedure was recorded then the bleed was classified as upper gastrointestinal, else it was classified as a lower gastrointestinal bleed. Upper gastrointestinal procedures were defined using the following OCPS-4 (Office of Population Censuses Surveys Classification of Surgical Operations and Procedures fourth revision) codes: fibreoptic endoscopic extirpation of lesion of oesophagus (G14); diagnostic fibreoptic endoscopic examination of oesophagus (G16); other fibreoptic endoscopic extirpation of lesion of upper gastrointestinal tract (G42); fibreoptic endoscopic extirpation of lesion of upper gastrointestinal tract (G43); other therapeutic fibreoptic endoscopic operations on upper gastrointestinal tract (G44); diagnostic fibreoptic endoscopic examination of upper gastrointestinal tract (G45); oversew of blood vessel of duodenal ulcer (G52.3); endoscopic extirpation of lesion of duodenum (G54.1); diagnostic endoscopic examination of duodenum (G55); endoscopic extirpation of lesion of jejunum (G64.1); diagnostic endoscopic examination of jejunum (G65); endoscopic extirpation of lesion of ileum (G79.1); diagnostic endoscopic examination of ileum (G80).

Harper C, *et al. Heart* 2023; 109:1467–1472. doi: 10.1136/heartjnl-2023-322616

Supplemental table 3: Codes used to identify any serious vascular events in routine data

Outcome	ICD-10 codes
Non-fatal myocardial infarction	Acute myocardial infarction (I21); subsequent myocardial infarction (I22)
Non-fatal presumed ischemic stroke	Cerebral infarction (I63); stroke, not specified as haemorrhage or infarction (I64)
Vascular death excluding intracranial haemorrhage	Diseases of the circulatory system, excluding haemorrhagic stroke (I00:I52, I63:I99)
Transient ischemic attack	Transient cerebral ischemic attacks, excluding transient global amnesia (G45.0:G45.3, G45.8:G45.9)

ICD-10 = International Statistical Classification of Diseases and Related Health Problems (tenth revision).

Harper C, *et al. Heart* 2023; 109:1467–1472. doi: 10.1136/heartjnl-2023-322616

BMJ Publishing Group Limited (BMJ) disclaims all liability and responsibility arising from any reliance placed on this supplemental material which has been supplied by the author(s)

Supplemental table 4: Agreement of routine data versus adjudicated direct follow-up for any major bleeding, by subgroups

Subgroup	Outcome in both datasets	Outcome in routine data only	Outcome in adjudicated follow-up alone	No such outcome in either dataset	Sensitivity (95% CI)	Specificity (95% CI)	Kappa (95% CI)	Heterogeneity test*
**Age (years)**								
<63	125 (1.6%)	113 (1.5%)	103 (1.3%)	7289 (95.5%)	54.8% (48.4%-61.3%)	98.5% (98.2%-98.8%)	0.52 (0.46-0.58)	
≥63	193 (2.5%)	168 (2.1%)	138 (1.8%)	7351 (93.6%)	58.3% (53.0%-63.6%)	97.8% (97.4%-98.1%)	0.54 (0.49-0.59)	0.71
**Sex**								
Male	207 (2.1%)	195 (2.0%)	152 (1.6%)	9130 (94.3%)	57.7% (52.5%-62.8%)	97.9% (97.6%-98.2%)	0.53 (0.48-0.57)	
Female	111 (1.9%)	86 (1.5%)	89 (1.5%)	5510 (95.1%)	55.5% (48.6%-62.4%)	98.5% (98.1%-98.8%)	0.54 (0.48-0.61)	0.67
**Vascular risk score****								
Low	88 (1.4%)	72 (1.1%)	71 (1.1%)	6033 (96.3%)	55.3% (47.6%-63.1%)	98.8% (98.5%-99.1%)	0.54 (0.47-0.61)	
Medium	132 (2.0%)	129 (2.0%)	95 (1.5%)	6192 (94.6%)	58.1% (51.7%-64.6%)	98.0% (97.6%-98.3%)	0.52 (0.46-0.58)	
High	98 (3.7%)	80 (3.0%)	75 (2.8%)	2415 (90.5%)	56.6% (49.3%-64.0%)	96.8% (96.1%-97.5%)	0.53 (0.46-0.60)	0.94
**Country**								
England	296 (2.1%)	266 (1.9%)	210 (1.5%)	13188 (94.5%)	58.5% (54.2%-62.8%)	98.0% (97.8%-98.3%)	0.54 (0.50-0.58)	
Other UK	22 (1.4%)	15 (1.0%)	31 (2.0%)	1452 (95.5%)	41.5% (28.2%-54.8%)	99.0% (98.5%-99.5%)	0.47 (0.32-0.62)	0.43
**All participants**	**318 (2.1%)**	**281 (1.8%)**	**241 (1.6%)**	**14640 (94.6%)**	**56.9% (52.8%-61.0%)**	**98.1% (97.9%-98.3%)**	**0.53 (0.49-0.57)**	

Percentages in parentheses are % of total number of ASCEND participants. *Heterogeneity test compares kappa statistics between subgroups. **We categorised the predicted 5-year risk of serious vascular event without the use of aspirin or fatty acids as follows: low risk as less than 5%, moderate risk as 5% to less than 10%, and high risk as 10% or more. CI = Confidence interval.

Harper C, *et al. Heart* 2023; 109:1467–1472. doi: 10.1136/heartjnl-2023-322616

Supplemental table 5: Comparison of routine data event date versus adjudicated direct follow-up

	Difference between event date in routine data and adjudicated direct follow-up					
**Outcome**	**Exact match**	**1-7 days**	**8-30 days**	**31-90 days**	**91-180 days**	**>180 days**
Intracranial haemorrhage	58 (66.7%)	22 (25.3%)	4 (4.6%)	1 (1.1%)	0 (0.0%)	2 (2.3%)
Sight-threatening bleeding in eye	10 (20.4%)	2 (4.1%)	4 (8.2%)	8 (16.3%)	3 (6.1%)	22 (44.9%)
Serious gastrointestinal bleeding	102 (77.9%)	14 (10.7%)	1 (0.8%)	2 (1.5%)	1 (0.8%)	11 (8.4%)
Upper bleeding	68 (76.4%)	9 (10.1%)	1 (1.1%)	2 (2.2%)	1 (1.1%)	8 (9.0%)
Lower bleeding	24 (80.0%)	3 (10.0%)	0 (0.0%)	0 (0.0%)	0 (0.0%)	3 (10.0%)
Unspecified bleeding	2 (100.0%)	0 (0.0%)	0 (0.0%)	0 (0.0%)	0 (0.0%)	0 (0.0%)
Perforation	3 (75.0%)	1 (25.0%)	0 (0.0%)	0 (0.0%)	0 (0.0%)	0 (0.0%)
Other major bleeding	29 (56.9%)	10 (19.6%)	1 (2.0%)	0 (0.0%)	3 (5.9%)	8 (15.7%)
Epistaxis	18 (78.3%)	3 (13.0%)	0 (0.0%)	0 (0.0%)	2 (8.7%)	0 (0.0%)
Haemoptysis	0 (0.0%)	0 (0.0%)	1 (100.0%)	0 (0.0%)	0 (0.0%)	0 (0.0%)
Haematuria	7 (50.0%)	3 (21.4%)	0 (0.0%)	0 (0.0%)	1 (7.1%)	3 (21.4%)
Vaginal bleeding	1 (100.0%)	0 (0.0%)	0 (0.0%)	0 (0.0%)	0 (0.0%)	0 (0.0%)
Unspecified bleeding	4 (44.4%)	3 (33.3%)	0 (0.0%)	0 (0.0%)	0 (0.0%)	2 (22.2%)
**Any major bleeding**	**190 (59.7%)**	**45 (14.2%)**	**13 (4.1%)**	**12 (3.8%)**	**7 (2.2%)**	**51 (16.0%)**

Percentages in parentheses are % of total number of participants where there was agreement between routine data and adjudicated follow-up. If a participant had more than one event occur during the follow-up period, we selected the earliest date.

Harper C, *et al. Heart* 2023; 109:1467–1472. doi: 10.1136/heartjnl-2023-322616

Supplemental table 6: Agreement of routine data versus adjudicated direct follow-up, by routine data algorithm

Routine data algorithm	Outcome in both datasets	Outcome in routine data only	Outcome in adjudicated follow-up alone	No such outcome in either dataset	Sensitivity (95% CI)	Specificity (95% CI)	Kappa (95% CI)
**No algorithm applied to routine data**							
Any bleeding code on hospital record	415 (2.7%)	1275 (8.2%)	144 (0.9%)	13646 (88.2%)	74.2% (70.6%-77.9%)	91.5% (91.0%-91.9%)	0.33 (0.30-0.37)
**One-factor-severity algorithms applied to routine data**							
Bleeding code as primary diagnosis	357 (2.3%)	778 (5.0%)	202 (1.3%)	14143 (91.4%)	63.9% (59.9%-67.8%)	94.8% (94.4%-95.1%)	0.39 (0.36-0.43)
Bleeding code in 1st/2nd position	389 (2.5%)	1017 (6.6%)	170 (1.1%)	13904 (89.8%)	69.6% (65.8%-73.4%)	93.2% (92.8%-93.6%)	0.36 (0.33-0.40)
Emergency admission	387 (2.5%)	537 (3.5%)	172 (1.1%)	14384 (92.9%)	69.2% (65.4%-73.1%)	96.4% (96.1%-96.7%)	0.50 (0.46-0.54)
Nights in hospital >1	326 (2.1%)	506 (3.3%)	233 (1.5%)	14415 (93.1%)	58.3% (54.2%-62.4%)	96.6% (96.3%-96.9%)	0.44 (0.41-0.48)
Nights in hospital >0	381 (2.5%)	622 (4.0%)	178 (1.1%)	14299 (92.4%)	68.2% (64.3%-72.0%)	95.8% (95.5%-96.2%)	0.46 (0.43-0.50)
**Two-factor-severity algorithms applied to routine data**							
Primary diagnosis + emergency	330 (2.1%)	288 (1.9%)	229 (1.5%)	14633 (94.5%)	59.0% (55.0%-63.1%)	98.1% (97.8%-98.3%)	0.54 (0.50-0.58)
1st/2nd diagnosis + emergency	366 (2.4%)	366 (2.4%)	193 (1.2%)	14555 (94.0%)	65.5% (61.5%-69.4%)	97.5% (97.3%-97.8%)	0.55 (0.51-0.59)
Primary diagnosis + nights >0	318 (2.1%)	281 (1.8%)	241 (1.6%)	14640 (94.6%)	56.9% (52.8%-61.0%)	98.1% (97.9%-98.3%)	0.53 (0.49-0.57)
Primary diagnosis + nights >1	274 (1.8%)	217 (1.4%)	285 (1.8%)	14704 (95.0%)	49.0% (44.9%-53.2%)	98.5% (98.4%-98.7%)	0.51 (0.46-0.55)
1st/2nd diagnosis + nights >0	352 (2.3%)	407 (2.6%)	207 (1.3%)	14514 (93.8%)	63.0% (59.0%-67.0%)	97.3% (97.0%-97.5%)	0.51 (0.48-0.55)
1st/2nd diagnosis + nights >1	300 (1.9%)	310 (2.0%)	259 (1.7%)	14611 (94.4%)	53.7% (49.5%-57.8%)	97.9% (97.7%-98.2%)	0.49 (0.45-0.53)
**Three-factor-severity algorithms applied to routine data**							
Primary + nights >0 + emergency	309 (2.0%)	235 (1.5%)	250 (1.6%)	14686 (94.9%)	55.3% (51.2%-59.4%)	98.4% (98.2%-98.6%)	0.54 (0.50-0.58)
1st/2nd + nights >0 + emergency	344 (2.2%)	307 (2.0%)	215 (1.4%)	14614 (94.4%)	61.5% (57.5%-65.6%)	97.9% (97.7%-98.2%)	0.55 (0.51-0.59)
Primary + nights >1 + emergency	266 (1.7%)	196 (1.3%)	293 (1.9%)	14725 (95.1%)	47.6% (43.4%-51.7%)	98.7% (98.5%-98.9%)	0.50 (0.46-0.55)
1st/2nd + nights >1 + emergency	293 (1.9%)	251 (1.6%)	266 (1.7%)	14670 (94.8%)	52.4% (48.3%-56.6%)	98.3% (98.1%-98.5%)	0.51 (0.47-0.56)
**Routine data algorithm selected for analyses***							
**Primary diagnosis + nights >0**	**318 (2.1%)**	**281 (1.8%)**	**241 (1.6%)**	**14640 (94.6%)**	**56.9% (52.8%-61.0%)**	**98.1% (97.9%-98.3%)**	**0.53 (0.49-0.57)**

Percentages in parentheses are % of total number of ASCEND participants. *All algorithms presented were considered, selection of the main algorithm for major bleeding in routine data was conducted by clinicians when blinded to the results of the randomised comparison. Selection were based on agreement statistics, simplicity of algorithm, and a definition of major bleeding broadly similar to ASCEND (see online supplemental figure 2). 1st/2nd = Bleeding code in 1st or 2nd position. CI = Confidence interval. Emergency = Emergency admission. Nights = Nights in hospital. Primary = Bleeding code as primary diagnosis.

Supplemental table 7: Agreement between routine data and adjudicated direct follow-up, by subcomponents of the major bleeding outcome

Outcome	Outcome in both datasets	Outcome in routine data only	Outcome in adjudicated follow-up alone	No such outcome in either dataset	Sensitivity (95% CI)	Specificity (95% CI)	Kappa (95% CI)
Intracranial haemorrhage	87 (0.6%)	50 (0.3%)	13 (0.1%)	15330 (99.0%)	87.0% (80.4%-93.6%)	99.7% (99.6%-99.8%)	0.73 (0.67-0.80)
Sight-threatening bleeding in eye	49 (0.3%)	48 (0.3%)	72 (0.5%)	15311 (98.9%)	40.5% (31.7%-49.2%)	99.7% (99.6%-99.8%)	0.45 (0.35-0.54)
Serious gastrointestinal bleeding	131 (0.8%)	96 (0.6%)	107 (0.7%)	15146 (97.8%)	55.0% (48.7%-61.4%)	99.4% (99.2%-99.5%)	0.56 (0.50-0.62)
Upper bleeding	89 (0.6%)	66 (0.4%)	63 (0.4%)	15262 (98.6%)	58.6% (50.7%-66.4%)	99.6% (99.5%-99.7%)	0.58 (0.50-0.65)
Lower bleeding	30 (0.2%)	39 (0.3%)	50 (0.3%)	15361 (99.2%)	37.5% (26.9%-48.1%)	99.7% (99.7%-99.8%)	0.40 (0.28-0.52)
Unspecified bleeding	2 (<0.1%)	0 (0%)	6 (<0.1%)	15472 (99.9%)			
Perforation	4 (<0.1%)	7 (<0.1%)	2 (<0.1%)	15467 (99.9%)			
Other major bleeding	51 (0.3%)	113 (0.7%)	66 (0.4%)	15250 (98.5%)	43.6% (34.6%-52.6%)	99.3% (99.1%-99.4%)	0.36 (0.26-0.45)
Epistaxis	23 (0.1%)	14 (0.1%)	16 (0.1%)	15427 (99.7%)			
Haemoptysis	1 (<0.1%)	5 (<0.1%)	3 (<0.1%)	15471 (99.9%)			
Haematuria	14 (0.1%)	43 (0.3%)	26 (0.2%)	15397 (99.5%)			
Vaginal bleeding	1 (<0.1%)	21 (0.1%)	6 (<0.1%)	15452 (99.8%)			
Unspecified bleeding	9 (0.1%)	34 (0.2%)	18 (0.1%)	15419 (99.6%)			
**Any major bleeding**	**318 (2.1%)**	**281 (1.8%)**	**241 (1.6%)**	**14640 (94.6%)**	**56.9% (52.8%-61.0%)**	**98.1% (97.9%-98.3%)**	**0.53 (0.49-0.57)**

Percentages in parentheses are % of total number of ASCEND participants. Due to low event numbers agreement statistics were not able to be calculated for most of the serious gastrointestinal and other major bleeding sub-components. Sensitivity and specificity statistics calculated using adjudicated direct follow-up as the reference dataset. CI = Confidence interval.

Harper C, *et al. Heart* 2023; 109:1467–1472. doi: 10.1136/heartjnl-2023-322616

Supplemental table 8: Major bleeding events identified in adjudicated direct follow-up alone or routine data only

	Events in adjudicated direct follow-up alone						Events in routine data only				
	Information from routine data*						Information from mail-based direct follow-up*				
**Outcome**	**Secondary diagnosis**	**No nights in hospital**	**Secondary diagnosis & no nights**	**Non-bleeding hospitalisation recorded**	**No hospitalisation recorded**	**Total**	**Refuted after adjudication**	**Minor bleed reported within 90 days**	**Non-bleeding hospitalisation reported**	**No hospitalisation reported**	**Total**
Intracranial haemorrhage	-	-	-	8 (61.5%)	5 (38.5%)	13	0 (0.0%)	-	13 (26.0%)	37 (74.0%)	50
Sight-threatening bleeding in eye	-	-	-	17 (23.6%)	55 (76.4%)	72	1 (2.1%)	0 (0.0%)	1 (2.1%)	46 (95.8%)	48
Serious gastrointestinal bleeding	34 (31.8%)	11 (10.3%)	1 (0.9%)	52 (48.6%)	9 (8.4%)	107	2 (2.1%)	1 (1.0%)	13 (13.5%)	80 (83.3%)	96
Other major bleeding	18 (27.3%)	10 (15.2%)	2 (3.0%)	25 (37.9%)	11 (16.7%)	66	8 (7.1%)	8 (7.1%)	16 (14.2%)	81 (71.7%)	113
**Any major bleeding**	**52 (21.6%)**	**19 (7.9%)**	**3 (1.2%)**	**93 (38.6%)**	**74 (30.7%)**	**241**	**11 (3.9%)**	**10 (3.6%)**	**37 (13.2%)**	**223 (79.4%)**	**281**

Percentages in parentheses are % of total number of ASCEND participants with an outcome in either adjudicated follow-up alone or routine data only. *Only events occurring within 90 days of the reported major bleeding event were used for these analyses.

Harper C, *et al. Heart* 2023; 109:1467–1472. doi: 10.1136/heartjnl-2023-322616

Supplemental table 9: Comparison of estimated treatment effects between adjudicated direct follow-up and routine data follow-up for the aspirin randomised comparison

	Rate ratios (95% confidence interval)		
**Outcome**	**Adjudicated direct follow-up**	**Routine data follow-up**	**Difference in point estimate***
Intracranial haemorrhage	1.22 (0.82-1.81)	1.24 (0.89-1.74)	+0.02 (-0.33, +0.35)
Sight-threatening bleeding in eye	0.89 (0.62-1.27)	0.90 (0.60-1.34)	+0.01 (-0.37, +0.39)
Serious gastrointestinal bleeding	1.36 (1.05-1.75)	1.41 (1.09-1.83)	+0.05 (-0.29, +0.40)
Other major bleeding	1.70 (1.18-2.44)	1.08 (0.79-1.46)	-0.62 (-1.25, -0.08)
**Any major bleeding**	**1.29 (1.09-1.52)**	**1.21 (1.03-1.41)**	**-0.08 (-0.28, +0.12)**

Log-rank methods were used to calculate the rate ratio and 95% confidence intervals. *Bootstrap methods were used to calculate the 95% confidence intervals for the difference in rate ratios between adjudicated follow-up and routine data. These methods used 1000 resamplings, with replacement, where the difference in point estimate was recalculated in each bootstrap sample.

Supplemental table 10: Effect of allocation to aspirin versus placebo on any major bleeding, by source of outcome data

	Outcome in both datasets			Outcome in adjudicated follow-up alone			Outcome in routine data only			All routine data and adjudicated follow-up events*		
**Outcome**	**Aspirin**	**Placebo**	**Rate ratio (95% CI)**	**Aspirin**	**Placebo**	**Rate ratio (95% CI)**	**Aspirin**	**Placebo**	**Rate ratio (95% CI)**	**Aspirin**	**Placebo**	**Rate ratio (95% CI)**
Intracranial haemorrhage	48	39	1.23 (0.81-1.87)	7	6	1.16 (0.39-3.45)	28	22	1.27 (0.73-2.21)	83	67	1.24 (0.90-1.70)
Sight-threatening bleeding in eye	20	29	0.69 (0.40-1.21)	37	35	1.06 (0.67-1.68)	26	22	1.18 (0.67-2.08)	83	85	0.98 (0.72-1.32)
Serious gastrointestinal bleeding	80	51	1.56 (1.11-2.19)	57	50	1.14 (0.78-1.67)	53	43	1.23 (0.82-1.84)	189	143	1.32 (1.07-1.64)
Other major bleeding	30	21	1.42 (0.82-2.46)	44	22	1.95 (1.20-3.16)	55	58	0.95 (0.66-1.37)	127	95	1.34 (1.03-1.74)
**Any major bleeding**	**178**	**140**	**1.27 (1.02-1.58)**	**136**	**105**	**1.30 (1.01-1.67)**	**149**	**132**	**1.13 (0.89-1.43)**	**460**	**369**	**1.25 (1.09-1.44)**

Log-rank methods were used to calculate the rate ratio and 95% confidence intervals. *Events identified in routine data only did not undergo adjudication and were excluded if within 90 days of an adjudicator refuted major bleeding event. Observed absolute effects of aspirin versus placebo on major bleeding for adjudicated direct follow-up and routine data combined were +8.4 per 5000 person-years (mean standard error ± 2.6). CI = Confidence interval.

Supplemental table 11: Effect of allocation to aspirin versus placebo on serious gastrointestinal and other major bleeding, by routine data algorithm

	Serious gastrointestinal bleeding			Other major bleeding		
**Routine data algorithm**	**Aspirin (n=7,740)**	**Placebo (n=7,740)**	**Rate ratio (95% CI)**	**Aspirin (n=7,740)**	**Placebo (n=7,740)**	**Rate ratio (95% CI)**
**No algorithm applied to routine data**						
Any bleeding code on hospital record	350 (4.5%)	341 (4.4%)	1.03 (0.88-1.19)	474 (6.1%)	437 (5.6%)	1.09 (0.95-1.24)
**One-factor-severity algorithms applied to routine data**						
Bleeding code as primary diagnosis	225 (2.9%)	212 (2.7%)	1.06 (0.88-1.28)	260 (3.4%)	264 (3.4%)	0.98 (0.83-1.17)
Bleeding code in 1st/2nd position	288 (3.7%)	279 (3.6%)	1.03 (0.88-1.22)	368 (4.8%)	329 (4.3%)	1.12 (0.97-1.30)
Emergency admission	196 (2.5%)	174 (2.2%)	1.13 (0.92-1.38)	202 (2.6%)	190 (2.5%)	1.06 (0.87-1.30)
Nights in hospital >1	175 (2.3%)	141 (1.8%)	1.24 (1.00-1.55)	177 (2.3%)	164 (2.1%)	1.08 (0.87-1.33)
Nights in hospital >0	208 (2.7%)	173 (2.2%)	1.20 (0.98-1.47)	248 (3.2%)	220 (2.8%)	1.13 (0.94-1.35)
**Two-factor-severity algorithms applied to routine data**						
Primary diagnosis + emergency	131 (1.7%)	105 (1.4%)	1.25 (0.97-1.61)	91 (1.2%)	81 (1.0%)	1.12 (0.83-1.52)
1st/2nd diagnosis + emergency	165 (2.1%)	129 (1.7%)	1.28 (1.02-1.61)	128 (1.7%)	115 (1.5%)	1.11 (0.87-1.43)
Primary diagnosis + nights >0	133 (1.7%)	94 (1.2%)	1.41 (1.09-1.83)	85 (1.1%)	79 (1.0%)	1.08 (0.79-1.46)
Primary diagnosis + nights >1	111 (1.4%)	71 (0.9%)	1.55 (1.16-2.08)	48 (0.6%)	47 (0.6%)	1.02 (0.68-1.53)
1st/2nd diagnosis + nights >0	168 (2.2%)	125 (1.6%)	1.34 (1.07-1.69)	151 (2.0%)	119 (1.5%)	1.27 (1.00-1.61)
1st/2nd diagnosis + nights >1	140 (1.8%)	96 (1.2%)	1.45 (1.13-1.88)	94 (1.2%)	77 (1.0%)	1.22 (0.90-1.65)
**Three-factor-severity algorithms applied to routine data**						
Primary + nights >0 + emergency	123 (1.6%)	85 (1.1%)	1.44 (1.10-1.89)	63 (0.8%)	58 (0.7%)	1.09 (0.76-1.55)
1st/2nd + nights >0 + emergency	155 (2.0%)	109 (1.4%)	1.42 (1.11-1.81)	96 (1.2%)	83 (1.1%)	1.16 (0.86-1.55)
Primary + nights >1 + emergency	102 (1.3%)	64 (0.8%)	1.58 (1.17-2.14)	42 (0.5%)	36 (0.5%)	1.17 (0.75-1.82)
1st/2nd + nights >1 + emergency	128 (1.7%)	82 (1.1%)	1.55 (1.18-2.03)	67 (0.9%)	56 (0.7%)	1.19 (0.84-1.70)
**Routine data algorithm selected for analyses***						
**Primary diagnosis + nights >0**	**133 (1.7%)**	**94 (1.2%)**	**1.41 (1.09-1.83)**	**85 (1.1%)**	**79 (1.0%)**	**1.08 (0.79-1.46)**

Log-rank methods were used to calculate the rate ratio and 95% confidence intervals. *All algorithms presented were considered, selection of the main algorithm for major bleeding in routine data was conducted by clinicians when blinded to the results of the randomised comparison. Selection were based on agreement statistics, simplicity of algorithm, and a definition of major bleeding broadly similar to ASCEND (see online supplemental figure 2). 1st/2nd = Bleeding code in 1st or 2nd position. CI = Confidence interval. Emergency = Emergency admission. Nights = Nights in hospital. Primary = Bleeding code as primary diagnosis.

Harper C, *et al. Heart* 2023; 109:1467–1472. doi: 10.1136/heartjnl-2023-322616

Supplemental table 12: Comparison of bleeding categories between pre and post adjudicated direct follow-up

	Bleeding category after adjudication			
**Bleeding category before adjudication**	**Major bleeding**	**Minor bleeding**	**No bleeding**	**Total**
Major bleeding (including all reported eye bleeds)	**505 (41.6%)**	496 (40.8%)	214 (17.6%)	**1215**
Minor bleeding (excluding eye bleeds)	24 (2.7%)	**801 (91.2%)**	53 (6.0%)	**878**
No bleeding	30 (0.2%)	70 (0.5%)	13287 (99.3%)	**13387**
**Total**	**559**	**1367**	**13554**	**15480**

Percentages in parentheses are % of total number of ASCEND participants with the bleeding category before adjudication. Each participant's records were searched for their first major bleeding event, if none identified then their records were searched for any minor bleeding code, if none identified then recorded as having no record of bleeding.

Supplemental table 13: Comparison of bleeding categories between pre and post adjudicated direct follow-up, by components of the serious gastrointestinal bleeding outcome

Bleeding category before adjudication	Bleeding category after adjudication						
	**Upper bleeding**	**Lower bleeding**	**Unspecified bleeding**	**Perforation**	**Minor bleeding**	**No bleeding**	**Total**
Upper bleeding	**135 (83.3%)**	3 (1.9%)	0 (0.0%)	0 (0.0%)	6 (3.7%)	18 (11.1%)	**162**
Lower bleeding	5 (3.4%)	**71 (47.7%)**	0 (0.0%)	0 (0.0%)	62 (41.6%)	11 (7.4%)	**149**
Unspecified bleeding	8 (53.3%)	0 (0.0%)	**6 (40.0%)**	0 (0.0%)	0 (0.0%)	1 (6.7%)	**15**
Perforation	1 (12.5%)	0 (0.0%)	0 (0.0%)	**5 (62.5%)**	0 (0.0%)	2 (25.0%)	**8**

Percentages in parentheses are % of total number of ASCEND participants with the bleeding category before adjudication.

Supplemental table 14: Comparison of bleeding categories between pre and post adjudicated follow-up, by components of the other major bleeding outcome

Bleeding category	Bleeding category after adjudication							
**before adjudication**	**Epistaxis**	**Haemoptysis**	**Haematuria**	**Vaginal bleeding**	**Unspecified bleeding**	**Minor bleeding**	**No bleeding**	**Total**
Epistaxis	**34 (48.6%)**	0 (0.0%)	1 (1.4%)	0 (0.0%)	0 (0.0%)	32 (45.7%)	3 (4.3%)	**70**
Haemoptysis	0 (0.0%)	**1 (33.3%)**	0 (0.0%)	0 (0.0%)	0 (0.0%)	2 (66.7%)	0 (0.0%)	**3**
Haematuria	0 (0.0%)	0 (0.0%)	**31 (23.8%)**	0 (0.0%)	0 (0.0%)	92 (70.8%)	7 (5.4%)	**130**
Vaginal bleeding	0 (0.0%)	0 (0.0%)	0 (0.0%)	**10 (13.9%)**	0 (0.0%)	58 (80.6%)	4 (5.6%)	**72**
Unspecified bleeding	0 (0.0%)	0 (0.0%)	0 (0.0%)	0 (0.0%)	**15 (36.6%)**	10 (24.4%)	16 (39.0%)	**41**

Percentages in parentheses are % of total number of ASCEND participants with the bleeding category before adjudication.

Harper C, *et al. Heart* 2023; 109:1467–1472. doi: 10.1136/heartjnl-2023-322616

Supplemental figure 1: Flow diagram of post-hoc analyses in the ASCEND trial

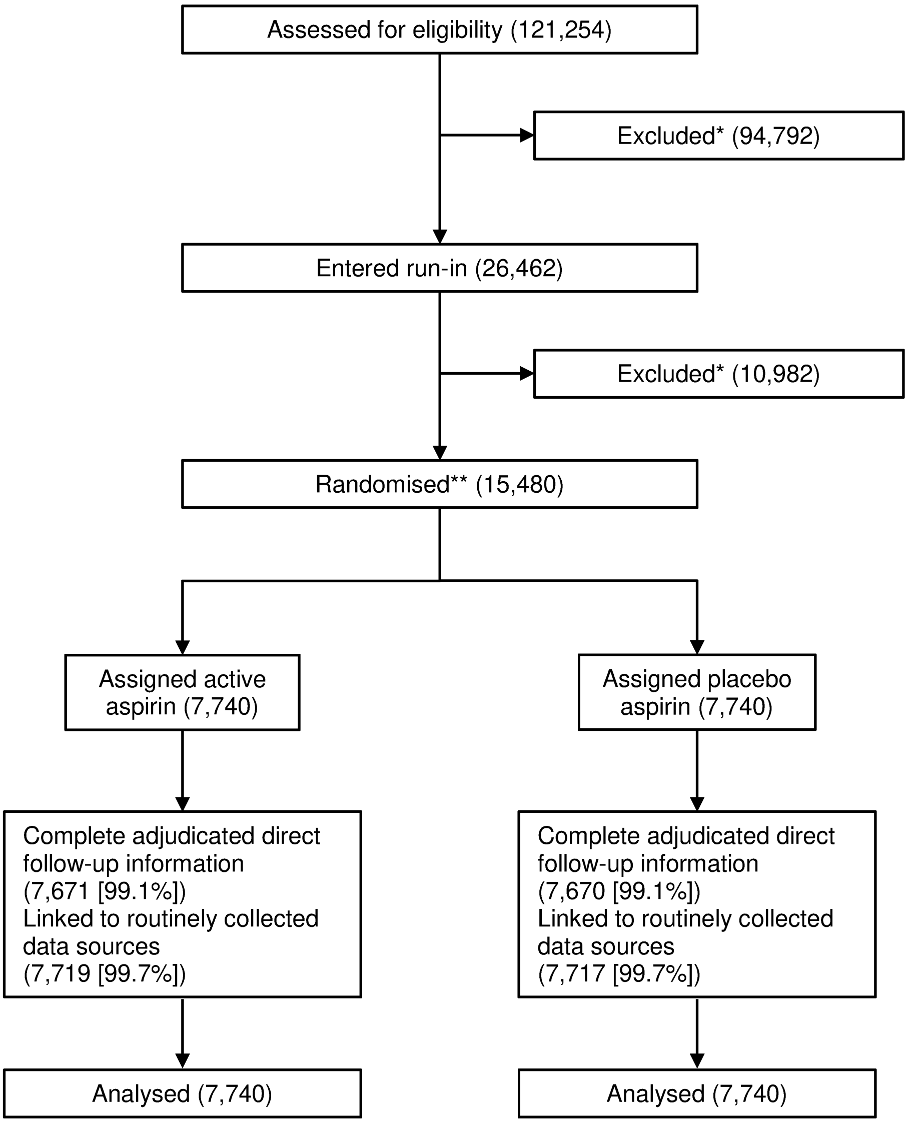

*A complete breakdown of exclusions can be found in ASCEND's main publications. **Omega-3 fatty acids randomised comparison not included in these post-hoc analyses.

Supplemental figure 2: Bleeding severity criteria for ASCEND adjudicated direct follow-up

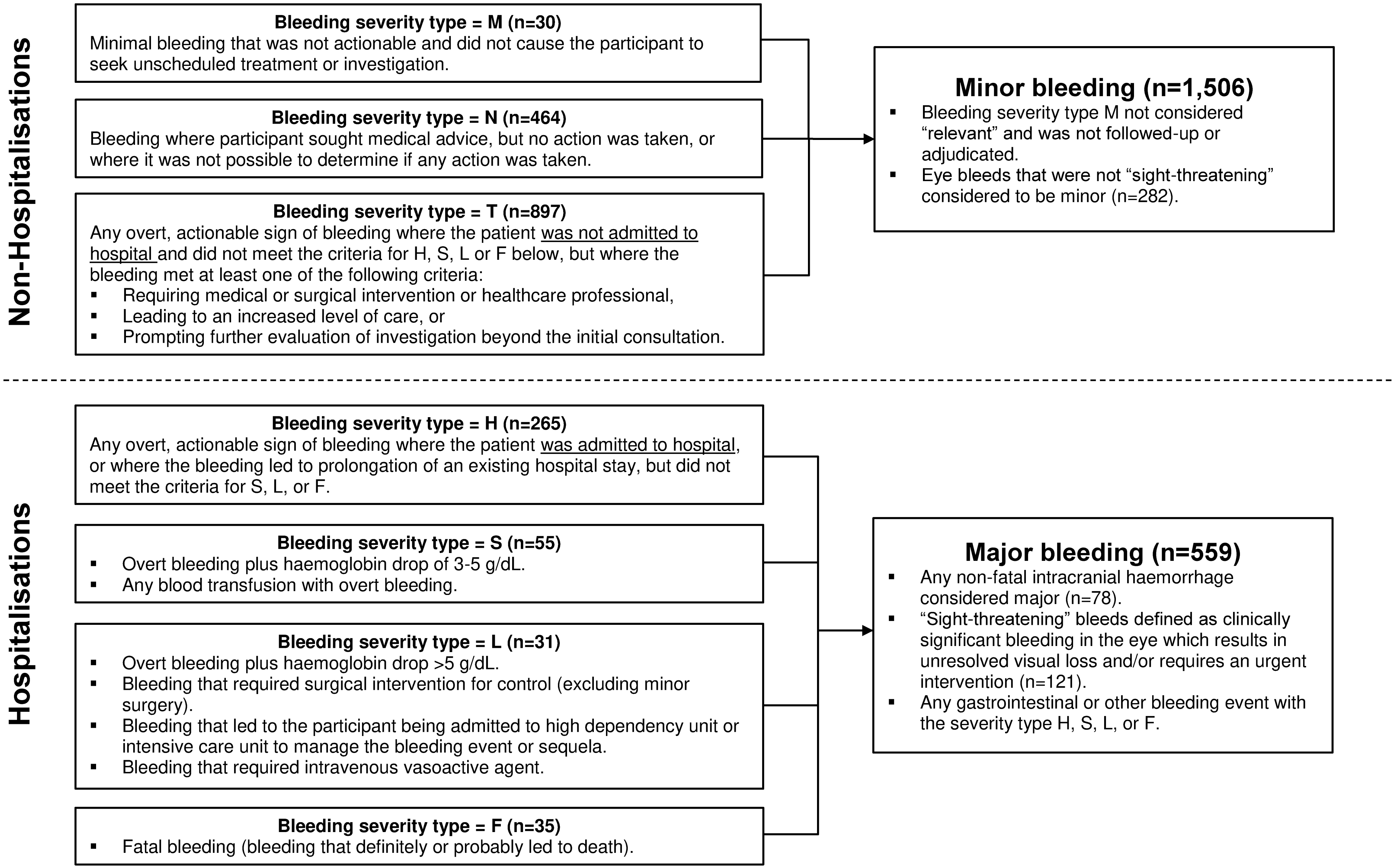

Bleeding severity criteria in ASCEND based on a modified version of the BARC bleeding definition (Bleeding Academic Research Consortium; *Circulation*. 2011;123:2736-2747); where BARC type 1 is broadly equivalent to ASCEND types M and N; BARC type 2 to ASCEND types T and H; BARC type 3a to ASCEND type S; BARC types 3b-4 to ASCEND type L; and BARC type 5 to ASCEND type F.

Supplemental figure 3: Sources of major bleeding events in ASCEND

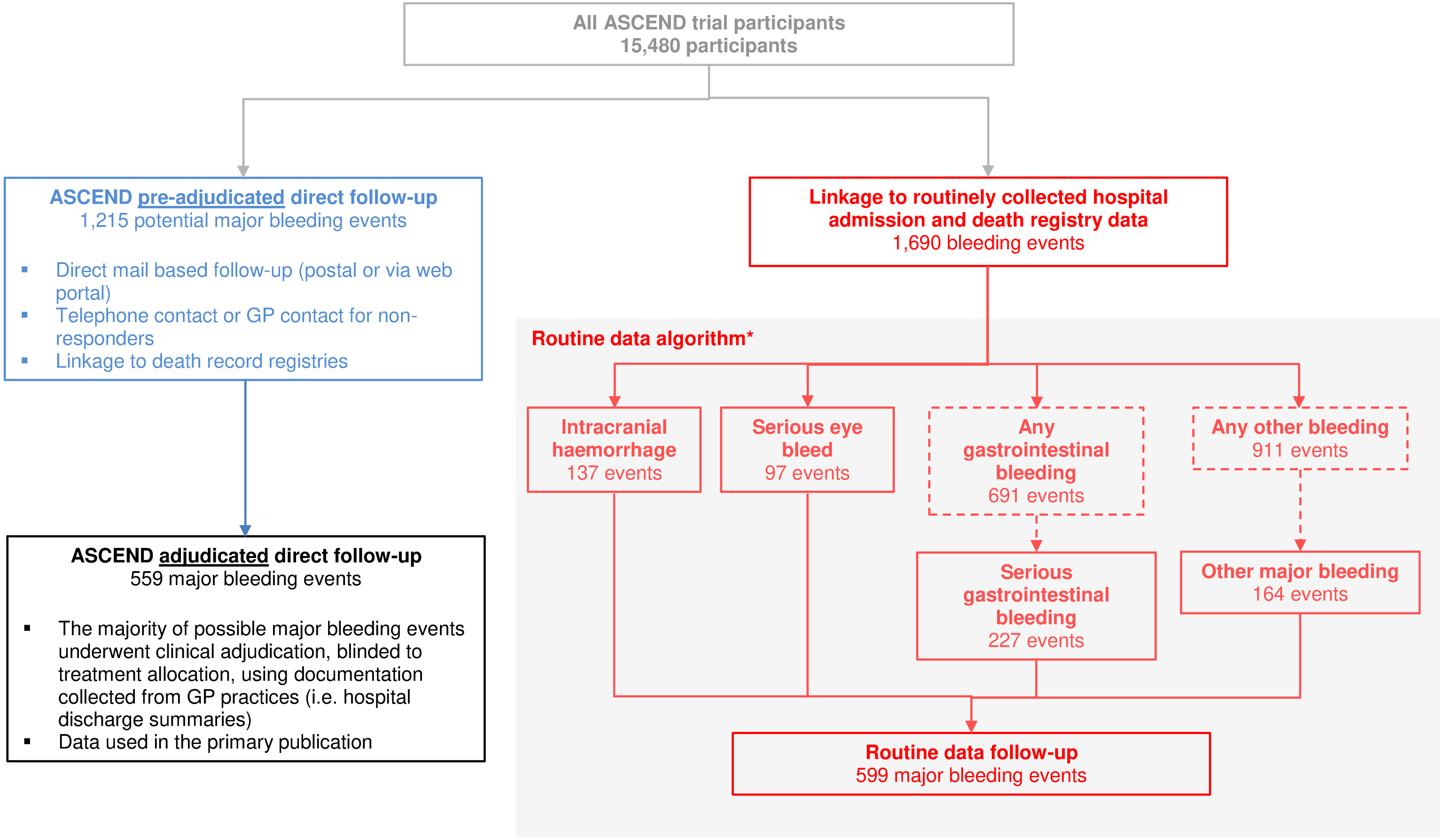

*For any intracranial haemorrhage or serious eye bleed, the routine data algorithm defined this as a code in any diagnostic position (i.e. primary or secondary diagnosis); while for gastrointestinal or other bleeding to be classified as major, records were restricted to bleeding codes in the first (i.e. primary) diagnostic position and patients having stayed at least one night in hospital (online supplemental table 2).

Harper C, *et al. Heart* 2023; 109:1467–1472. doi: 10.1136/heartjnl-2023-322616

Supplemental figure 4: Effect of allocation to aspirin versus placebo on any major bleeding, by routine data algorithm

Routine data algorithms:
Considered
Selected for analyses*

Adjudicated direct follow-up

	Aspirin (n=7,740)	Placebo (n=7,740)	Rate ratio (95% CI)	Absolute effect
**No algorithm applied to routine data**				
Any bleeding code on hospital record	866 (11.2%)	824 (10.6%)	1.05 (0.96-1.16)	+ 4.0 ± 3.8
**One-factor-severity algorithms applied to routine data**				
Bleeding code as primary diagnosis	574 (7.4%)	561 (7.2%)	1.02 (0.91-1.15)	+ 1.2 ± 3.1
Bleeding code in 1st/2nd position	726 (9.4%)	680 (8.8%)	1.07 (0.96-1.19)	+ 4.4 ± 3.5
Emergency admission	484 (6.3%)	440 (5.7%)	1.10 (0.97-1.26)	+ 4.1 ± 2.7
Nights in hospital >1	444 (5.7%)	388 (5.0%)	1.15 (1.00-1.31)	+ 7.0 ± 2.9
Nights in hospital >0	539 (7.0%)	464 (6.0%)	1.17 (1.03-1.32)	+ 5.1 ± 2.6
**Two-factor-severity algorithms applied to routine data**				
Primary diagnosis + emergency	333 (4.3%)	285 (3.7%)	1.17 (1.00-1.37)	+ 4.4 ± 2.2
1st/2nd diagnosis + emergency	396 (5.1%)	336 (4.3%)	1.18 (1.02-1.37)	+ 5.5 ± 2.4
Primary diagnosis + nights >0	327 (4.2%)	272 (3.5%)	1.21 (1.03-1.41)	+ 5.0 ± 2.2
Primary diagnosis + nights >1	271 (3.5%)	220 (2.8%)	1.23 (1.03-1.47)	+ 4.6 ± 2.0
1st/2nd diagnosis + nights >0	422 (5.5%)	337 (4.4%)	1.26 (1.09-1.45)	+ 7.8 ± 2.5
1st/2nd diagnosis + nights >1	340 (4.4%)	270 (3.5%)	1.26 (1.08-1.48)	+ 6.3 ± 2.2
**Three-factor-severity algorithms applied to routine data**				
Primary + nights >0 + emergency	298 (3.9%)	246 (3.2%)	1.21 (1.03-1.44)	+ 4.7 ± 2.1
1st/2nd + nights >0 + emergency	359 (4.6%)	292 (3.8%)	1.23 (1.06-1.44)	+ 6.1 ± 2.3
Primary + nights >1 + emergency	257 (3.3%)	205 (2.6%)	1.26 (1.05-1.51)	+ 4.7 ± 1.9
1st/2nd + nights >1 + emergency	304 (3.9%)	240 (3.1%)	1.27 (1.07-1.50)	+ 5.8 ± 2.1
**Routine data algorithm selected for analyses***				
Primary diagnosis + nights >0	327 (4.2%)	272 (3.5%)	1.21 (1.03-1.41)	+ 5.0 ± 2.2
**Adjudicated direct follow-up (as reported by main publication)**				
**Any major bleeding**	**314 (4.1%)**	**245 (3.2%)**	**1.29 (1.09-1.52)**	**+ 6.3 ± 2.1**

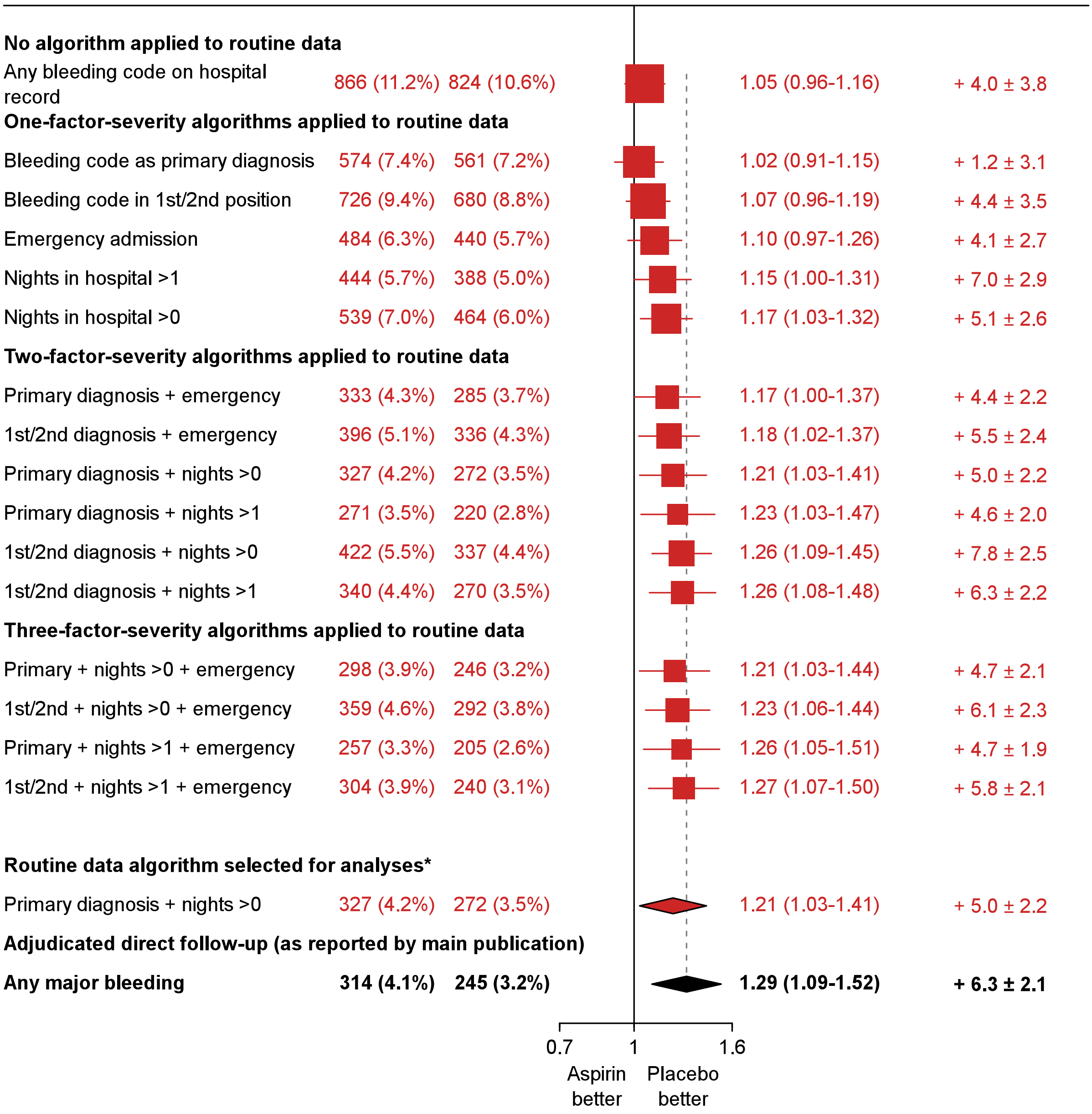

Log−rank methods were used to calculate the rate ratio and 95% confidence intervals. Absolute effects expressed as numbers of events per 5000 person−years, plus−minus values are means ± standard error. *All algorithms presented were considered, selection of algorithm for major bleeding in routine data was conducted by clinicians when blinded to results of the randomised comparison. Selection were based on agreement statistics, simplicity of algorithm, and a definition of major bleeding broadly similar to ASCEND (see online supplemental figure 2). 1st/2nd = Bleeding code in 1st or 2nd position. CI = Confidence interval. Emergency = Emergency admission. Nights = Nights in hospital. Primary = Bleeding code as primary diagnosis.
