# Supplementary material for: Reliability of major bleeding events in UK routine data versus clinical trial adjudicated follow-up data
Source: Heart. 2023 Jun 3;109(19):1467–72. doi: 10.1136/heartjnl-2023-322616 (PMC10511984; doi:10.1136/heartjnl-2023-322616)
Supplement: Supplementary data [file heartjnl-2023-322616supp002.pdf]

Supplemental Methods

Adjudication in the ASCEND trial

Study clinicians blind to treatment allocation adjudicated the primary and secondary outcomes. For non-fatal vascular and bleeding outcomes, the coordinating centre requested documentation (e.g. hospital discharge summaries) from primary care. In a small number of cases where participants were lost to follow-up, routine hospital admission data were used to ascertain 18 serious vascular events and 24 major bleeds. For fatal outcomes, the primary source of information was the Office for National Statistics death certification data, which included deaths occurring in the UK both in and out of hospital. The reported primary and subsidiary causes of death were then reviewed by study clinicians along with available hospital admissions data and any information from the trial questionnaires. The published results were based on all confirmed and unrefuted serious vascular and major bleeding event reports (i.e. a reported event for which supporting documentation could not be obtained [i.e. was unrefuted] was counted in the analysis). Over 90% of the serious vascular events and major bleeding events included in the analysis were confirmed by adjudication. Details of the adjudication definitions (including how deaths were ascribed to their underlying cause using information from review of other clinical information alongside death certificates) are provided in the Supplement accompanying the main publication.[1]

Routine data sources

During follow-up, death records were obtained from the Office for National Statistics via NHS Digital for England/Wales[2] and National Health Scotland Central Register[3]. These data included date of death, underlying and other contributing causes of death. Participants were also linked to their routinely collected hospital admission records; these data were obtained from NHS Digital (Hospital Episode Statistics Admitted Patient Care)[4] for England, Public Health Scotland (Scottish Morbidity Records 01)[5], and the Welsh SAIL Databank (Patient Episode Database for Wales)[6]. Information used for these analyses included: the primary and any secondary admission diagnoses; any operations and procedures; admission method; admission date; and the number of nights the participant stayed in hospital. Linkage between the trial participants and routine datasets were carried out by the national registries, but was not possible for 44 participants (44/15480 [0.3%]) residing in Northern Ireland, among which only one serious vascular event and no major bleeding event occurred

Harper C, *et al. Heart* 2023; 109:1467–1472. doi: 10.1136/heartjnl-2023-322616

during follow-up. At the time of this study participants were not linked to their primary care general practice data.

Derivation of routine data algorithm

Potential indicators of event severity that were considered based on previous observational studies[7-11] included: bleeding in the primary (i.e. first) or second diagnostic position on the admission record (where up to twenty diagnoses can be recorded), patients having stayed at least one or two nights in hospital, and admission via emergency means. Severity factors that were not considered due to missing or incomplete information on the routinely collected hospitalisation record included blood transfusions, medical or surgical interventions, and falls in haemoglobin. Fifteen algorithms were derived using either one-severity-factor (e.g. emergency admission), two-severity-factors in combination (e.g. primary diagnosis and emergency admission), or three-severity-factors. For each possible algorithm, kappa, sensitivity and specificity statistics were calculated using adjudicated direct follow-up as the comparator. Of the fifteen possible algorithms, one was selected for the primary analyses by clinicians before any randomised comparisons were re-run. The selection was based on the agreement statistics, simplicity of the algorithm, and a definition broadly similar to the ASCEND trial criteria set out in online supplemental figure 2. For any intracranial haemorrhage or serious eye bleed, the selected algorithm defined this as an ICD-10 code in any diagnostic position (i.e. primary or secondary diagnosis); while for gastrointestinal or other bleeding to be classified as major, records were restricted to bleeding codes in the first (i.e. primary) diagnostic position and patients having stayed at least one night in hospital (full details of the selected algorithm can be found in online supplemental table 2).

Statistical analyses

Overall levels of agreement between routine data and adjudicated direct-participant follow-up were estimating using the kappa statistic.[12] Agreement between subgroups were assessed using heterogeneity testing, including analyses split by median age (<63 vs ≥63-years), sex (male vs female), vascular risk score (low vs medium vs high [see ASCEND data analysis plan[13]]), and country of residence (England vs Other UK). Where there was agreement between two sources of outcome data (i.e. outcomes in both datasets), the event dates were compared. Differences were presented as: exact match (same day), 1-7 days, 8-30 days, 31-90 days, 91-180 days, and >180 days. Where an event was reported in adjudicated direct-participant follow-up alone, routine data was searched to

Harper C, *et al. Heart* 2023; 109:1467–1472. doi: 10.1136/heartjnl-2023-322616

identify whether there was a corresponding hospitalization (within 90 days of the adjudicated event date) or death record for the same participant. A similar process was carried out to search adjudicated data for events recorded solely in routine data.

Supplemental References

1. ASCEND Study Collaborative Group, Bowman L, Mafham M, et al. Effects of Aspirin for Primary Prevention in Persons with Diabetes Mellitus. *N Engl J Med*. Oct 18 2018;379(16):1529-1539. doi:10.1056/NEJMoa1804988
2. Civil Registration - Deaths. Health Data Research Innovation Gateway. https://www.healthdatagateway.org/dataset/050163dc-1728-4ac5-a7d9-4dd3ca0ca12a. Accessed March 1, 2021.
3. National Records of Scotland (NRS) - Deaths Data. Health Data Research Innovation Gateway. https://www.healthdatagateway.org/dataset/e600dae2-a83c-4b7a-8d23-af4ac31ca374. Accessed March 1, 2021.
4. Hospital Episode Statistics Admitted Patient Care. Health Data Research Innovation Gateway. https://web.www.healthdatagateway.org/dataset/2b6409db-a669-4bef-9fd5-39c2b6f8d5e9. Accessed March 1, 2021.
5. General Acute Inpatient and Day Case - Scottish Morbidity Record (SMR01). Health Data Research Innovation Gateway. https://www.healthdatagateway.org/dataset/98cda353-0011-45b2-80ca-4ed24cd084bf. Accessed March 1, 2021.
6. Patient Episode Dataset for Wales. Health Data Research Innovation Gateway. https://www.healthdatagateway.org/dataset/4c33a5d2-164c-41d7-9797-dc2b008cc852. Accessed March 1, 2021.
7. Pasea L, Chung SC, Pujades-Rodriguez M, et al. Bleeding in cardiac patients prescribed antithrombotic drugs: electronic health record phenotyping algorithms, incidence, trends and prognosis. *BMC Med*. Nov 20 2019;17(1):206. doi:10.1186/s12916-019-1438-y
8. Pasea L, Chung SC, Pujades-Rodriguez M, et al. Personalising the decision for prolonged dual antiplatelet therapy: development, validation and potential impact of prognostic models for cardiovascular events and bleeding in myocardial infarction survivors. *Eur Heart J*. Apr 7 2017;38(14):1048-1055. doi:10.1093/eurheartj/ehw683
9. Timmis A, Rapsomaniki E, Chung SC, et al. Prolonged dual antiplatelet therapy in stable coronary disease: comparative observational study of benefits and harms in unselected versus trial populations. *Bmj*. Jun 22 2016;353:i3163. doi:10.1136/bmj.i3163
10. Hippisley-Cox J, Coupland C. Predicting risk of upper gastrointestinal bleed and intracranial bleed with anticoagulants: cohort study to derive and validate the QBleed scores. *BMJ*. Jul 28 2014;349:g4606. doi:10.1136/bmj.g4606
11. Button LA, Roberts SE, Evans PA, et al. Hospitalized incidence and case fatality for upper gastrointestinal bleeding from 1999 to 2007: a record linkage study. *Alimentary pharmacology & therapeutics*. Jan 2011;33(1):64-76. doi:10.1111/j.1365-2036.2010.04495.x
12. Cohen J. A Coefficient of Agreement for Nominal Scales. *Educational and Psychological Measurement*. 1960/04/01 1960;20(1):37-46. doi:Doi 10.1177/001316446002000104
13. Bowman L, Mafham M, Stevens W, et al. ASCEND: A Study of Cardiovascular Events iN Diabetes: Characteristics of a randomized trial of aspirin and of omega-3 fatty acid supplementation in 15,480 people with diabetes. *Am Heart J*. Apr 2018;198:135-144. doi:10.1016/j.ahj.2017.12.006
